# Development of Serological Assays and Seroprevalence Studies of the New Coronavirus 2019 (COVID-19): Reports from Saudi Arabia

**DOI:** 10.3390/healthcare9121730

**Published:** 2021-12-14

**Authors:** Thamir A. Alandijany, Arwa A. Faizo

**Affiliations:** 1 Special Infectious Agents Unit, King Fahd Medical Research Center, King Abdulaziz University, P.O. Box 128442, Jeddah 21362, Saudi Arabia; 2 Department of Medical Laboratory Technology, Faculty of Applied Medical Sciences, King Abdulaziz University, P.O. Box 80324, Jeddah 21589, Saudi Arabia

**Keywords:** COVID-19, SARS-CoV-2, epidemiology, immunoassays, ELISA, micro-neutralization assay, Saudi Arabia, serology, seroprevalence

## Abstract

Serological assays are valuable tools for tracking COVID-19 spread, estimation of herd immunity, and evaluation of vaccine effectiveness. Several reports from Saudi Arabia describe optimized in-house protocols that enable detection of SARS-CoV-2 specific antibodies and measurement of their neutralizing activity. Notably, there were variations in the approaches utilized to develop and validate these immunoassays in term of sample size, validation methodologies, and statistical analyses. The developed enzyme-linked immunoassays (ELISAs) were based on the viral full-length spike (S), S1 subunit, and nucleocapsid (NP), and enabled detection of IgM and/or IgG. ELISAs were evaluated and validated against a microneutralization assay utilizing a local SARS-CoV-2 clinical isolate, FDA-approved commercially available immunoassays, and/or real-time polymerase chain reaction (RT-PCR). Overall, the performance of the described assays was high, reaching up to 100% sensitivity and 98.9% specificity with no cross-reactivity with other coronaviruses. In-house immunoassays, along with commercially available kits, were subsequently applied in a number of sero-epidemiological studies aiming to estimate sero-positivity status among local populations including healthcare workers, COVID-19 patients, non-COVID-19 patients, and healthy blood donors. The reported seroprevalence rates differed widely among these studies, ranging from 0.00% to 32.2%. These variations are probably due to study period, targeted population, sample size, and performance of the immunoassays utilized. Indeed, lack of sero-positive cases were reported among healthy blood donors during the lockdown, while the highest rates were reported when the number of COVID-19 cases peaked in the country, particularly among healthcare workers working in referral hospitals and quarantine sites. In this review, we aim to (1) provide a critical discussion about the developed in-house immunoassays, and (2) summarize key findings of the sero-epidemiological studies and highlight strengths and weaknesses of each study.

## 1. Introduction

The World Health Organization (WHO) declared the Coronavirus Disease of 2019 (COVID-19), caused by the novel severe acute respiratory syndrome coronavirus 2 (SARS-CoV-2), a pandemic on 11 March 2020 [1,2]. The first cases of COVID-19 were reported from Wuhan, China in late December 2019 [3]. Since then, the infection has spread worldwide, leading to more than 100 million cases and 2 million deaths approximately one year after reporting the first case [4]. For better or worse, officials in the affected countries have responded to this crisis with different strategies. In Saudi Arabia, policymakers have been proactive in their response to COVID-19 and initiated control measures even before reporting the country’s first case [5,6]. These measures include evacuation of all Saudi citizens from Wuhan, China, suspension of international flights from and to more than 20 COVID-19-hit countries, including China, and suspension of Umrah (a shorter version of Muslim pilgrimage to Makkah) and visit to the Holy Mosque [5,6]. Following detection of COVID-19 cases in the country, further management plans were implemented such as curfew and lockdown of major cities, closure of mosques and educational institutes, banning sport and social events, and provision of free-of-charge healthcare to COVID-19 patients, including those illegally present in the country [5,6]. The Ministry of Health announced a three-stage plan to return to “normal” life starting from 28 May to 21 June 2020. Although many COVID-19 restrictions have been relaxed since then, social and physical distancing and facemask wearing are still enforced by the Saudi government, even among those who received COVID-19 vaccines. COVID-19 vaccines have been offered in Saudi Arabia free-of-charge to both citizens and residents starting December 2020. As of 26 October 2021, more than 60% of population in Saudi Arabia received two doses of COVID-19 vaccine. This should substantially contribute to minimizing viral spread among the population. As of May 17, Saudi Arabia decided to open land, sea, and air borders. With the lack of specific antiviral therapy and limited supply of vaccination to some countries, in addition to the emergence of COVID-19 variants that may confer resistance to the current vaccines, SARS-CoV-2 might continue to spread among the population in the next few months [7]. Continuous monitoring of COVID-19 cases by molecular detection of viral nucleic acid, such as reverse-transcriptase polymerase chain reaction (RT-PCR), was vital for COVID-19 surveillance [8,9]. With the presence of asymptomatic but infectious COVID-19 individuals, immunoassays that enable detection of SARS-CoV-2 specific antibodies are valuable tools for identifying these cases [10]. Indeed, it is important at this stage to conduct epidemiological seroprevalence studies to provide an estimation of the extent of viral spread and investigate the current herd immunity status of Saudi population. It is also key to utilize reliable techniques and methodologies to avoid under or over-estimation of the current seroprevalence status. Several reports from different research groups (including ours) in the country have addressed this issue with various study populations (healthcare workers versus healthy individuals), sample size (ranged from hundreds to thousands), and methodologies utilized (in-house versus commercial immunoassays). In this review, we aim to deliver a balanced evaluation of the in-house immunoassays developed in Saudi Arabia, summarize the key findings of the local COVID-19 sero-epidemiological studies, and highlight the strengths and weaknesses of each study. This critical discussion can be valuable not only for COVID-19 research but also for other infectious diseases.

## 2. Development of Serological Assays for COVID-19

Many labs in Saudi Arabia possess FDA-approved serological assays that enable sensitive and specific detection of SARS-CoV-2 antibodies. However, accumulating evidence has raised concerns with regards to the performance of some of these assays [11,12,13,14,15]. Sensitivity and specificity of these commercial assays, in some cases, were below 70% [13]. Hence, some research groups decided to develop their own in-house or semi-in-house assays in order to minimize the potential of misdiagnosis and “false” reporting [16,17,18,19]. Development of in-house assays was also necessary during the early phase of COVID-19 pandemic when many countries experienced major delivery interruption of local and international shipments.

The developed assays were mostly validated against a micro-neutralization assay (MN assay; also called serum neutralization assay), reverse-transcriptase polymerase chain reaction (RT-PCR), or other FDA-approved commercial immunoassays [16,17,18,19]. A summary of the developed COVID-19 immunoassays, their performance, and their corresponding validation methodologies are shown in Table 1. Each approach has its pros and cons, and thereby it is important to discuss them.

Our research group has published two ELISA protocols that enable detection of human IgG antibodies to SARS-CoV-2 spike (S) and nucleocapsid (NP) proteins [16,17]. The optimized antigen coating concentration, and sample and conjugate dilution were determined. Both assays offered 100% sensitivity, >98% specificity and agreement, and high reproducibility and overall accuracy [16,17]. The performance of these assays was validated against an MN assay and some FDA-approved commercial kits [16,17]. Although the MN assay represents the gold standard for neutralizing antibody detection, this assay requires biosafety containment Level 3 and, thereby, it is not available for some researchers [11,15]. Furthermore, recent evidence demonstrated cross-neutralization activity of antibodies raised to other coronaviruses against SARS-CoV-2 [20,21]. To overcome this issue, the developed assays were evaluated with sera containing antibodies to SARS-CoV-2, Middle East respiratory syndrome coronavirus (MERS-CoV) and human coronavirus HKU1 (HCoV-HKU1), and demonstrated specificity to SARS-CoV-2 antibodies [16,17]. However, cross-reactivity evaluation with other coronaviruses or respiratory viruses was not assessed due to lack of samples. The reasons for the minimal false positive results (>98% specificity) reported with these assays remained unidentified.

An independent research group described high-performance ELISA protocols for detection of IgM and IgG antibodies utilizing the SARS-CoV-2 S1 subunit and NP proteins [18]. The assay sensitivity and specificity were evaluated against RT-PCR results, which might be suboptimal given that not all COVID-19 patients mount an antibody response [18,22,23,24,25]. The assay performance was also monitored over four weeks following the onset of symptoms and showed high overall performance. However, it is important to note that the number of serum samples utilized were sometimes very limited (e.g., five samples only for the 4th week of symptoms-onset) [18]. Importantly, the assessment of the potential cross-reactivity with several other coronaviruses (MERS-CoV, hCoV-OC43, and hCoV-HKU1, hCoV-NL63 and hCoV-229) was examined and the developed ELISAs demonstrated high specificity [18]. However, the authors pointed out that comprehensive conclusion with regards to their IgM ELISAs cross-reactivity with other coronaviruses could not be drawn due to lack of samples [18].

Another local article reported optimized S-based ELISA protocols for detection of anti-SARS-CoV-2 IgM and IgG [19]. The optimization process relied on (1) positive serum samples obtained from current COVID-19 patients and recovered individuals based on their RT-PCR results, and (2) negative serum samples obtained from the pre-pandemic era [19]. The author concluded that the described protocols are sensitive and specific. Furthermore, high concordance with commercially available chemiluminescent immunoassay (CLIA) was found, and cross-reactivity was not detected with antibodies directed to other respiratory viruses (including influenza viruses and MERS-CoV) [19]. However, we could not find the relevant statistical analyses (sensitivity, specificity, agreement, Coefficient of variation for inter- and intra-assays) in this article. Data related to cross-reactivity evaluation and concordance with CLIA were not shown. In addition, there was a lack of sufficient information about the CLIA utilized in the study. Correspondingly, conducting an independent evaluation about the performance of these optimized ELISAs was challenging.

Apart from ELISAs, research groups have published some protocols for assessing the neutralizing activity of COVID-19 antibodies. Utilizing local SARS-CoV-2 clinical isolate (SARS-CoV-2/human/SAU/85791C/2020) (Genbank accession number MT630432.1), an in-house micro-neutralization assay (the gold standard) has been described [17,26]. Due to the requirement of biosafety containment Level 3 to handle the live virus, which might not be available to all researchers, pseudovirus neutralization assays using vesicular stomatitis virus (VSV)-based pseudoviruses and lentivirus-based pseudoviruses expressing SARS-CoV-2 S protein were stated to overcome this issue [27,28]. Providing quantitative and semiquantitative results was an added value for these assay [27,28].

At the international level, similar studies describing COVID-19 immunoassays have been reported from several research groups [29,30,31,32,33,34]. Most of these immunoassays were based on SARS-CoV-2 full-length S, NP, or fragments of S (e.g., receptor binding domain). In some cases, assays were optimized not only for detection of IgM and IgG, but also for IgA [33]. Validation of results was also evaluated against RT-PCR, the MN assay, and commercially available kits [29,30,31,32,33,34]. Herein, we review several in-house immunoassays developed in Saudi Arabia. Although researchers have utilized various techniques to validate their assays, the assay optimized conditions are very similar with overall reliable performance. These studies represent another forward step toward technology localization and knowledge transfer in the country. Further, the in-house ELISAs and neutralization assays developed in Saudi Arabia, along with commercially available immunoassays, were subsequently utilized in several local epidemiological studies providing valuable information with regard to the seroprevalence status of COVID-19 among the population of Saudi Arabia.

**Table 1:** Summary of in-house COVID-19 immunoassays developed in Saudi Arabia. The assay principle, the evaluation methods, and the performance indicators are shown.

Type of Assay	Evaluation Methods	Sensitivity	Specificity	Other Indicators of Assay Performance	Ref.
Full length S-based IgG ELISA	Microneutralization assay	100%	98.4%	Agreement: 98.8% Accuracy: AUC = 0.9996 ± 0.0003; 95% CI of 0.99 to 1.00 Reproducibility: <12% variation No cross-reactivity antibodies directed to with MERS-CoV and HCoV HKU1	[17]
NP-based IgG ELISA	Microneutralization assay, in-house S-based ELISA, and a commercial kit	100%	98.9%	Agreement: 98.9% Accuracy: AUC = 0.9998 ± 0.0002; 95% CI of 0.99 to 1.00 Reproducibility: <10% variation No cross-reactivity antibodies directed to with MERS-CoV and HCoV HKU1	[16]
ELISAs: S1-based IgM S1-based IgG NP-based IgM NP-based IgM	Real-time polymerase chain reaction (RT-PCR)	100% 100% 100% 60%	97.6% 97.6% 94.4% 91.2%	Accuracy: ranging from 0.886 ± 0.037 to 0.977 ± 0.015; 95% CI from 0.8122 1.00 Reproducibility: 5–10% variation No cross-reactivity with antibodies directed to MERS-CoV, HCoV HKU1, hCoV-OC43, hCoV-NL63 and the hCoV-229.	[18]
Full length S-based IgG ELISA	Real-time polymerase chain reaction (RT-PCR) and a commercial kit	Unknown	Unknown	No cross-reactivity with antibodies directed to MERS-CoV or influenzas viruses	[19]
Pseudo-virus neutralization assay (lentivirus-based expressing SARS-CoV-2 S protein)	Microneutralization assay	85.94%	100%	Offer quantitative results	[28]

## 3. Seroprevalence Status among Healthcare Workers

Many COVID-19 seroprevalence studies have been reported from Saudi Arabi. These studies substantially vary in terms of target population, sample size, immunoassays utilized, and findings. A summary of these studies is shown in Table 2. The first seroprevalence study reported from the country targeted healthcare workers, due to the nature of their work and their close contact to patients [35]. This multicenter seroprevalence study comprised relatively large number of participants (*n* = 12,621) and displayed the overall sero-status of COVID-19 among healthcare workers [35]. Moreover, a direct side-by-side comparison was conducted between COVID-19 referral hospitals and nonaffected hospitals. Sera were obtained from participants working in 85 health centers and hospitals across the nation between 20 May and 30 May 2020 [35]. Notably, this was only three months after reporting the country’s first case in March 2020 [5]. Initially, all serum samples were screened for the presence of IgG utilizing commercially available chemiluminescent microparticle immunoassay. The number of positive sera was 299. demonstrating an overall seroprevalence rate of 2.37% [35]. A variation in the seroprevalence rates between regions and cities was noticed ranging from 0% to 6.31% [35]. For unknown reasons, out of the 299 positive sera, only 100 were subjected to SARS-CoV-2 pseudo-typed viral particles neutralization, and 92% possessed neutralizing activity [35]. Hence, the neutralization status of the remaining 199 CLIA positive sera remained vague. Although important, participants’ details with regards to past COVID-19 diagnosis could not be found.

After publishing this paper, several other reports on the sero-status of COVID among healthcare workers started to appear [22,36,37]. Unlike the nationwide seroprevalence study discussed above, most of these studies were performed on healthcare workers from single hospitals with a relatively limited number of participants [22,36]. However, an added value for these studies was the diversity of serological assays utilized. For instance, a study from our research group conducted in-house ELISA and commercial CLIA in addition to micro-neutralization assays on all serum samples collected from June and July 2020 to determine their serostatuses, which enabled another level of confidence with regards to the reported findings [22]. This study demonstrated an overall seroprevalence rate of 6.3% with high concordance among results obtained from the three immunoassays [22]. Importantly, this study also identified asymptomatic previously undiagnosed cases, and showed evidence that not all recovered patients mounted an efficient neutralizing antibody response [22]. Information with regards to the intervals between sample collection and patients’ recovery was not provided [22].

Another study from our lab was performed on operating room and critical care staff and demonstrated a 12.2% sero-positivity rate [36]. The study period was between 9 August 2020 to 2 November 2020, which may explain the increase in the seroprevalence status [36]. This study also performed extensive statistical analyses to identify risk factors associated with increased risk of acquiring COVID-19 among healthcare workers (e.g., contact with a COVID-19 family member); such information is valuable for disease control and prevention [36]. Interestingly, being in close contact with COVID-19 patients in the hospital or performing intubation did not significantly affect the sero-status of healthcare workers [36]. Recently, a sero-epidemiological study that targeted healthcare workers during the epidemic peak in the country (from 29 June to 10 August 2020) reported remarkably high seroprevalence rate (32.2%) compared to other studies [37]. Interestingly, 88.3% of positive cases were never diagnosed with COVID-19, and about 62% did not report COVID-19 symptoms [37]. Participants were healthcare workers from referral hospitals and quarantine sites, which probably increased their exposure to the virus [37]. This study sheds light on the importance of continuous monitoring of the serostatus of population beside utilizing serology with molecular testing for COVID-19 diagnosis in order to identify asymptomatic individuals. However, it is important to note that all serum samples were collected from individuals residing in a single city (Jeddah, Saudi Arabia). Furthermore, the number of cases in this particular city peaked from May to June just before sample collection time (29 June to 10 August 2020) which leaves the epicenters for initial viral spread in this major Saudi city undefined.

**Table 2:** Summary of seroprevalence studies of COVID-19 in Saudi Arabia. The targeted study population, study period, immunoassays utilized, the seroprevalance rates, and other key findings are shown.

Study Population	Study Period	Methodologies	Sero-Prevalence Rate	Other Key Findings of the Studies	Ref.
Blood donors (*n* = 956) from a single center	1 January to 31 May 2020	(1) In-house SARS-CoV-2 S-based ELISA (2) In-house MN assay	0.00%	- A complete lack of sero-positive cases. - None of the particpants were previously diagnosed with COVID-19.	[38]
Blood donors (*n* = 837) from different cities/regions	20th to 25th May 2020	Commercially available NP-based electro-CLIA	1.4%	- There was variation in the seroprevalence rate (ranging from 0 to 8.1% between regions and/or cities) - Nationality and education levels significantly affected the serostatus	[39]
Healthcare workers (*n* = 12,621) from 85 hospitals	20 and 30 May 2020	(1) commercially available NP-based microparticle CLIA (2) SARS-CoV-2 pseudotyped viral particles (SARS2pp) neutralization assay	2.36%	There was variation in the seroprevalence rate (ranging from 0% to 6.31%) between regions and/or cities.	[35]
Blood donors (*n* = 1212)	mid-May and mid-July 2020	In-house SARS-CoV-2 S-based ELISA	19.31%	Blood group, but not age, significantly affected the serostatus.	[40]
Healthcare workers (*n* = 204) from a single hospital	June and July 2020	(1) In-house SARS-CoV-2 S-based ELISA (2) commercially available SARS-CoV-2 NP-based electro-CLIA, (3) In-house MN assay	6.3%	- High concordance between three immunoassays - Identification of seropositivity among previously undiagnosed cases. - Identification of recovered patients without mounting antibody response.	[22]
Healthcare workers (*n* = 693) from referral hospitals and quarantine sites	29 June to 10 August 2020	(1) In-house SARS-CoV-2 S- and NP-based ELISA (2) In-house Pseudovirus neutralization Assay	32.2%	- Most positive cases (88.3%) were previously undiagnosed with COVID-19 - 62.8% of sero-positive cases did not report any COVID-19 symptoms (asymptomatic)	[37]
Blood donors, non-COVID-19 patients, and HCW (*n* = 11,703) from different cities/regions	June to November 2020	In-house and commercially available SARS-CoV-2 S-based ELISA	10.9%	There was variation in the seroprevalence rate (ranging from 5.1 to 18.8% between regions and/or cities)	[41]
Healthcare workers (*n* = 319) from a single hospital	9 August 2020 to 2 November 2020	(1) In-house SARS-CoV-2 S-based ELISA (2) In-house MN assay	12.2%%	- Identification of seropositivity among previously undiagnosed cases. - Identifying contact with COVID-19 family member as a risk factor for acquiring the infection - Neither working in close contact with COVID-19 patients nor performing intubation significantly affected the serostatus.	[36]

## 4. Seroprevalence Status among the General Population

In addition to healthcare workers, seroprevalence was investigated among blood donors and non-COVID-19 patients in order to estimate the seropositivity status among the general public [38,40,41]. The first study investigated this issue among all blood donors who attended a single-donation center from January to May 2020 [38]. All of these donors were never diagnosed with COVID-19. Combining results obtained from in-house ELISA and MN assays, a lack of sero-positive cases was concluded [38]. Importantly, the study period covered two months prior to reporting the first COVID-19 case in Saudi Arabia plus three months when the number of daily cases peaked in the country [5]. This study was supported by nationwide studies [39,41]. Indeed, the seroprevalence was as low as 1.4% among blood donors who were not diagnosed with COVID-19 during the first months of the pandemic [39]. Nationality and education status of participants significantly affected the seroprevalence rate, being lower among Saudis and those with higher education [39]. Commercial CLIA with 99.8% sensitivity and 99.5% specificity was used [39]. Therefore, minimal probabilities of false positives and false negatives exist which may have contributed to the 1.4% seropositivity rate. Further, the neutralizing activity of the detected antibodies remain unknown. An independent nationwide study comprising more than 11 thousand participants demonstrated high overall seroprevalence (11%) among healthy blood donors and non-COVID-19 patients, reaching up to 20% in some cities [41]. This study also utilized some healthcare workers’ samples who were never diagnosed with COVID-19 but showed 7.5% sero-positive rate [41]. Sera were subjected to several ELISAs protocols in order to confirm these findings. In comparison with other studies, the seroprevalence rates was approximately 10 times higher [41]. Similar higher seroprevalence rate (19.31%) was reported among blood donors who attended a single donation center from mid-May to mid-July 2020 [40]. A likely explanation for these findings is the fact that sample collection was conducted at a later time than previous studies and following the relaxation of COVID-19 restriction [5]. In addition, these studies highlighted the variation in rates between different cities and populations within the same country. It is interesting, however, to note that none of the aforementioned studies could identify high risk groups to acquire COVID-19 just before and during the peak of COVID-19 cases in the country.

## 5. Conclusions

Great effort has been made toward developing serological assays for COVID-19 and conducting sero-epidemiolocal investigations in Saudi Arabia. The in-house immunoassays were optimized and validated utilizing different methodologies, but generally expressed high overall performance. Local sero-surveillance studies are key to accurately estimate the prevalence of populations that have been infected with SARS-CoV-2, particularly those infected but who remained asymptomatic. These studies can provide valuable information about “epicenters” of infection spread among societies, although there is a need for further investigations with regards to this matter in Saudi Arabia. Having been validated, a high-performance serology assay is of valuable utility in the current era of COVID-19 vaccines. Accumulating evidence demonstrates the efficiency and safety of COVID-19 vaccines. However, more studies are still required to draw comprehensive conclusions among various population who may respond differently to the vaccine due to various environmental (e.g., geographic location and weather), behavioral (e.g., smoking, physical activity, and quality of sleep), and nutritional factors (e.g., body mass index). There is currently a lack of local reporting about vaccine efficiency in Saudi Arabia, although it has been almost a year since the vaccine was introduced to the country. With more than 47.3 million doses (as of 30 November 2020) offered to the population of Saudi Arabia, serological assays will be key to assess vaccine effectiveness in inducing immunity.

## Data Availability

Not applicable.
